# Physical function and sex differences in radiographic axial spondyloarthritis: a cross-sectional analysis on Bath Ankylosing Spondylitis Functional Index

**DOI:** 10.1186/s13075-023-03173-w

**Published:** 2023-09-26

**Authors:** Magnus Hallström, Eva Klingberg, Anna Deminger, Jeannette Beckman Rehnman, Mats Geijer, Helena Forsblad-d’Elia

**Affiliations:** 1 Department of Rheumatology and Inflammation Research, Institute of Medicine, University of Gothenburg, Box 480, 405 30 Gothenburg, Sweden; 2 Department of Rheumatology, Region Västra Götaland, Sahlgrenska University Hospital, Gothenburg, Sweden; 3 Department of Public Health and Clinical Medicine, Section of Medicine, Umeå University, Umeå, Sweden; 4 Department of Radiology, Institute of Clinical Sciences, University of Gothenburg, Gothenburg, Sweden; 5 Department of Radiology, Region Västra Götaland, Sahlgrenska University Hospital, Gothenburg, Sweden; 6 Department of Clinical Sciences, Lund University, Lund, Sweden

**Keywords:** Spondyloarthritis, Ankylosing spondylitis, Functional performance, Radiography, Sex, Fatigue, Cross-sectional study

## Abstract

**Background:**

Physical function is an important determinant of health-related quality of life in radiographic axial spondyloarthritis patients (r-axSpA). To improve the basis of effective healthcare efforts, we aimed to investigate which demographic and disease-related factors that influence Bath Ankylosing Spondylitis Functional Index (BASFI) in r-axSpA patients overall and stratified by sex. Furthermore, we sought to explore differences between sexes regarding separate BASFI questions and also to explore which factors that may contribute to these differences.

**Methods:**

This observational cross-sectional study included patients fulfilling the modified New York criteria for Ankylosing Spondylitis. Patients were assessed with 66/68 joint count and Bath Ankylosing Spondylitis Metrology Index (BASMI) measurements. Lateral X-rays were performed for Modified Stoke Ankylosing Spondylitis Spinal Score (mSASSS). Bath Ankylosing Spondylitis Disease Activity Index (BASDAI), Ankylosing Spondylitis Disease Activity Score (ASDAS)-C-Reactive Protein (CRP), and BASFI were registered. Multivariable linear regression analyses were used to investigate which factors that associate with BASFI.

**Results:**

A total of 353 r-axSpA patients were included, mean age 52.2 ± 12.7 years, 62.3% males. No significant sex difference was seen in BASFI scores (2.7 ± 2.0 in males vs 2.9 ± 2.1 in females). Age, body mass index, ASDAS-CRP, BASMI or mSASSS, fatigue, and tenderness were found to associate independently with BASFI in different models (*R*^2^ 0.53–0.63). Investigation of separate BASFI questions revealed that the ability to look over shoulder was worse in males than females (mean 4.43 ± 3.37 vs 3.74 ± 3.06, *p* = 0.05) and most strongly correlated with mSASSS and BASMI among separate BASFI questions (*r* = 0.53, *p* < 0.001; *r* = 0.62, *p* < 0.001). The ability to climb stairs was worse in females than males (mean 2.49 ± 2.77 vs 1.54 ± 2.32, *p* < 0.001).

**Conclusions:**

No difference between male and female r-axSpA patients was seen in BASFI despite significant sex differences in BASMI, mSASSS, and CRP levels. Our results underline the impact of fatigue and tenderness on BASFI. The ability to climb stairs without a handrail was scored worse among females compared to males. Furthermore, the ability to look over the shoulder was worse in males than females and closely related to spinal mobility and structural spinal changes.

**Supplementary Information:**

The online version contains supplementary material available at 10.1186/s13075-023-03173-w.

## Background

Ankylosing spondylitis (AS), also known as radiographic axial spondyloarthritis (r-axSpA), is a rheumatic inflammatory condition which has a significant impact on the affected patients and on costs for the society [1, 2]. Physical function is an important determinant of health-related quality of life (HRQoL) in r-axSpA patients and is known to be influenced independently by disease activity and accumulated structural r-axSpA-related alterations in the spine [3, 4]. To evaluate physical ability, the Bath Ankylosing Spondylitis Functional Index (BASFI) was developed in 1994 [5]. BASFI consists of ten questions; eight specific questions regarding the level of difficulty to perform various physical tasks in everyday life and two questions reflecting the patient’s ability to cope with daily life. The BASFI instrument is widely used, is validated, quick, and easy to complete, and has been found to be reliable and sensitive to change [6, 7]. In addition, it is recommended for assessing physical function as one of the core outcomes in the recently updated Assessment of SpondyloArthritis international Society-Outcomes Measures in Rheumatology (ASAS-OMERACT) guidelines [8]. In recent years, accumulating evidence has identified several important differences between the male and female r-axSpA phenotypes. In particular, males are affected by more comprehensive radiographic spinal skeletal alterations than females [9, 10]. Regarding patient-reported outcome measures (PROMS) in r-axSpA, females tend to score higher in the Bath Ankylosing Spondylitis Disease Activity Index (BASDAI) than males while the Ankylosing Spondylitis Disease Activity Score (ASDAS) is reported without a clear difference between the sexes [11] and concerning BASFI, findings diverge in terms of a sex-difference or not [12–15]. Thorough analyses of the different BASFI questions in relation to sex in r-axSpA are overall lacking.

To improve personalized healthcare efforts in r-axSpA patients, increased knowledge of what factors influence male and female functional impairment is important. Therefore, we aimed to investigate which demographic and disease-related factors that influence physical impairment in r-axSpA patients overall and in addition, stratified by sex. Furthermore, we wanted to elucidate how the different BASFI questions related to sex and how this could be explained. As a secondary aim, we sought to explore if predictors of BASFI differed between two separate cohorts of patients with r-axSpA, one from western Sweden and the other from northern Sweden.

## Methods

### Patients

**Fig. 1:**
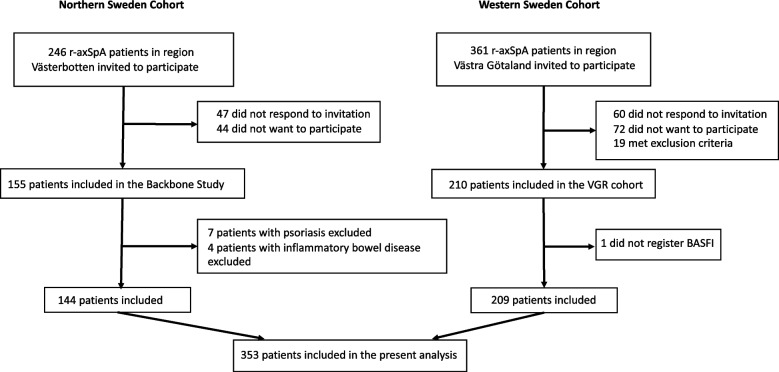
Flowchart of the inclusion process.* r-axSpA* radiographic axial spondyloarthritis

Patients with a diagnosis of AS (ICD-10 M45.9) were identified using the hospitals’ digital administrative systems in western Sweden (Sahlgrenska University Hospital (SU) and the county hospitals of Borås and Alingsås) and in Region Västerbotten of northern Sweden (University Hospital in Umeå (NUS), and the county hospitals of Skellefteå and Lycksele). The medical records of all patients were subsequently reviewed by a rheumatologist and only those fulfilling the modified New York criteria for AS were asked to participate in the study [16]. Recently, it was suggested that AS and r-axSpA can be used as interchangeable terms, and thus, we will use the name r-axSpA in this report [17]. Exclusion criteria were psoriasis, inflammatory bowel disease, dementia, pregnancy, having another rheumatic disease, and difficulty in understanding the Swedish language. As shown in the flowchart in Fig. 1, out of initially 607 invited participants, 107 did not respond to the invitation,116 did not want to participate, and 19 met exclusion criteria. Two hundred nine patients from western Sweden were enrolled in 2009 whereas 144 patients from northern Sweden were included during 2016–2017 as previously described in detail [18, 19]. Baseline inclusion data of 353 participants in the combined cohort were used in the current study. Written informed consent was obtained from all patients, and the study was approved by the regional ethical review boards in Gothenburg (597–08) and Umeå (2016/208–31), Sweden. The study was performed in accordance with the Declaration of Helsinki.

### Physical examination and patient reported outcome measures

At inclusion, the 66/68 joint count was assessed and mobility measures including the Bath Ankylosing Spondylitis Metrology Index (BASMI) were performed by experienced rheumatologists, as described previously [20, 21]. Weight and height were measured, and body mass index (BMI) was calculated. All patient-reported outcome measures (PROMs) were assessed at the same study visit, including BASFI and BASDAI [5, 22]. ASDAS was also assessed at inclusion in all patients except for those enrolled in 2009 where this outcome was calculated retrospectively [23]. In these instruments, higher values reflect worse outcomes. Furthermore, the patients answered questions regarding comorbidities, smoking habits, medication, and disease-related features such as hip arthritis.

### Radiography

Lateral x-rays of the cervical and lumbar spine were acquired according to the study protocol.

The radiographs were scored by the same experienced radiologist for cumulative AS-related spinal structural alterations using Modified Stoke Ankylosing Spondylitis Spinal Score (mSASSS) [24]. Intra-reader reliability of this specific radiologist has been previously assessed showing excellent agreement, intraclass correlation coefficient 0.98 (95% CI 0.96 to 0.99) [10].

### Laboratory tests

Blood samples were drawn for measurement of high sensitivity (hs) C-reactive protein (CRP) and erythrocyte sedimentation rate (ESR) and were consecutively analyzed with standard laboratory techniques. The presence of the human leukocyte antigen (HLA)-B27 was controlled in all patients.

### Statistics

Continuous variables are presented as mean and standard deviation (SD) or median and quartile 1 (Q_1_) and quartile 3 (Q_3_) where appropriate, while categorical variables are shown as numbers and percentages. An independent samples *t*-test was used to compare continuous variables, whereas the chi-squared test was used for dichotomous variables. ANOVA and the Tukey HSD test were applied when comparing several groups. For correlation, Pearson correlation analyses were used. To investigate which factors influence r-axSpA-related physical impairment, multivariable linear regression analyses were performed using the BASFI score as the outcome. Because the left-skewed distribution of BASFI caused heteroscedasticity, BASFI was stratified into 10-percentiles. Stratification of the outcome produced improved residual scatterplots and more robust results compared to log transformation. Percentile stratification was therefore also used in analyses of the individual BASFI questions. The inclusion of predictors was made by the enter method. Regression models were performed with all patients included and stratified by sex. As our secondary aim, and as a sensitivity analysis, the final models were repeated with the patients separated by geographical cohort. Independent variables were selected according to hypothetical reasoning and to what is previously known from the literature. For a full list of evaluated variables, see the correlation matrix in Supplementary Table 1, Additional file 1. Because BASDAI questions (QN) “overall level of fatigue/tiredness” (BASDAI QN1) and “discomfort from areas tender to touch” (BASDAI QN4) are not included in the ASDAS instrument, they were evaluated as covariates alongside ASDAS. In the initial, exploring models, 17 different demographic and disease-related variables were selected as predictors of BASFI. The total number of independent variables included in the initial sex-stratified models was reduced to maintain good prediction despite fewer cases. Male sex was coded as 1 and female sex as 2. The following independent factors that did not affect the model significantly were excluded from the final models: delay of diagnosis, swollen joint count 66, tender joint count 68, history of hip arthritis, history of peripheral arthritis, history of anterior uveitis, HLA-B27, ever/never smoker, and smoking packyears. Symptom duration was excluded due to strong collinearity with age (*r* > 0.7) whereas BASMI and mSASSS were analyzed in separate models, also because of collinearity (*r* > 0.7). To elucidate which factors influence the sex differences demonstrated in the current study, the procedure was repeated using the individual BASFI questions “look over your shoulder without turning the body” (BASFI question QN8) and “climbing 12–15 steps without using a handrail or walking aid” (BASFI QN7) as outcomes. These two variables were also skewed, and while a homoscedastic model could be achieved using BASFI QN8 grouped into 10-percentiles, BASFI QN7 had to be grouped into 20-percentiles. To shed light on the association between individual BASFI questions, BASMI, mSASSS, and the cervical portion of mSASSS, bivariate correlation analysis was performed using each individual BASFI item and mSASSS, cervical mSASSS, followed by BASMI. All analyses were performed using SPSS statistics v.27 (SPSS Inc., IBM corp., Armonk, NY, USA) and *p* ≤ 0.05 was considered statistically significant.

## Results

### Patients

Totally, 353 r-axSpA patients were included in the current analysis (Fig. 1) of which 220 (62.3%) were males (Table 1). The mean age was overall 52.2 ± 12.7 years, with a mean symptom duration of 27.1 ± 13.0 years. BMI was slightly higher among males compared to females. HLA-B27 was present in 91.8% and more prevalent among male than female patients (95.5% vs. 85.7%, *p* = 0.001). HsCRP was increased in males compared to females, whereas ESR was higher in females than males. Among clinical outcome measures, BASDAI was reported higher in females (4.0 ± 2.0 vs. 3.4 ± 2.1, *p* = 0.006), while BASMI was higher in males (3.7 ± 1.8 vs. 3.3 ± 1.4, *p* = 0.01). Tender joint count 68 was higher in females than in males. Furthermore, female participants had fewer radiographic spinal alterations overall and also in the cervical spine compared to males. No difference between sexes was seen regarding BASFI or ASDAS-CRP. The radiographs were acquired 36.0 ± 32.5 days (mean ± SD) after the clinical assessments. Concerning therapy, current medication was evenly distributed between sexes, and overall, 19.5% of patients were prescribed biological disease- modifying anti-rheumatic drugs (bDMARDs) (Table 1).

**Table 1:** Descriptive characteristics of 353 patients with radiographic axial spondyloarthritis

General characteristics				
	**All patients**	**Males**	**Females**	*p*-value
Sex	353	220 (62.3)	133 (37.7)	
Age, years	52.2 ± 12.7	51.7 ± 13.0	53.0 ± 12.2	0.4
BMI, kg/m^2^	26.8 ± 4.9	27.2 ± 5.0	25.9 ± 4.6	**0.008**
Current smoker	39 (11)	24 (10.9)	15 (11.3)	0.9
Ever smoker	168 (47.6)	103 (46.8)	65 (48.9)	0.7
r-axSpA variables				
Symptom duration, years	27.1 ± 13.0^a^	26.8 ± 12.8^d^	27.5 ± 13.4^e^	0.7
HLA-B27-positive	324 (91.8)	210 (95.5)	114 (85.7)	**0.001**
ESR, mm/h	14.6 ± 12.8, 11 (6, 19)	13.2 ± 12.3, 10 (5, 17)	16.8 ± 13.4, 14.0 (8, 23)	**0.009**
hsCRP, mg/L	5.4 ± 8.3, 2.8 (1, 6)^b^	6.1 ± 9.5, 3.0 (1, 7)	4.4 ± 5.9, 2.1 (1, 5)^e^	**0.04**
History of anterior uveitis	181 (51.2)	117 (53.2)	64 (48.1)	0.4
History of peripheral arthritis	206 (58.4)	118 (53.6)	88 (66.2)	**0.02**
History of coxitis	47 (13.3)	27 (12.3)	20 (15.0)	0.5
BASDAI, score	3.6 ± 2.0^a^	3.4 ± 2.1^f^	4.0 ± 2.0	**0.006**
ASDAS CRP, score	2.0 ± 0.9^b^	2.0 ± 0.9^ g^	2.1 ± 0.8	0.4
BASFI, score	2.8 ± 2.1^c^	2.7 ± 2.0^ g^	2.9 ± 2.1^e^	0.2
BASMI, score	3.5 ± 1.7	3.7 ± 1.8	3.3 ± 1.4	**0.01**
BASDAI QN1 Fatigue	4.6 ± 2.6	4.2 ± 2.5	5.2 ± 2.6	**0.001**
BASDAI QN4 Level of discomfort	3.0 ± 2.7^c^	2.6 ± 2.4^d^	3.7 ± 2.8	** < 0.001**
mSASSS, score	15.5 ± 19.7, 6 (0, 24)^c^	20.5 ± 21.6, 11.5 (2, 34)^d^	7.4 ± 12.5, 2.0 (0, 10)	** < 0.001**
mSASSS cervical, score	7.0 ± 11.2, 1.0 (0, 9)^c^	9.6 ± 12.5, 2.5 (0, 17)^d^	2.8 ± 6.9, 0.0 (0, 2)	** < 0.001**
Days to X-rays	36.0 ± 32.5, 27.5 (21, 41)^c^	38.7 ± 39.6, 27.0 (21, 40)^d^	31.4 ± 13.6, 28.0 (22, 41)	**0.007**
Swollen joint count 66	0.2 ± 0.9^b^	0.2 ± 1.0	0.1 ± 0.8^e^	0.9
Tender joint count 68	1.3 ± 4.9	0.4 ± 1.2	2.8 ± 7.5	** < 0.001**
Delay of diagnosis, years	9.2 ± 8.1^a^	8.0 ± 7.5^d^	11.3 ± 8.6^e^	** < 0.001**
csDMARD monotherapy	40 (11.3)	21 (9.5)	19 (14.3)	0.2
bDMARD monotherapy	29 (8.2)	20 (9.1)	9 (6.8)	0.4
csDMARD and bDMARD	40 (11.3)	29 (13.2)	11 (8.3)	0.2
NSAID, regular or on demand	279 (79.3)^b^	170 (77.3)	109 (82.0)^e^	0.2

Values are mean ± standard deviation, median (quartile (Q)_1_, Q_3_), or *n* (%). Independent samples *t*-test was used for continuous variables. Chi-squared test was used for dichotomous variables. Highlighted in bold are *p*-values ≤ 0.05

*BMI* body mass index, *r-axSpA* radiographic axial spondyloarthritis, *HLA-B27* human leukocyte antigen B27, *ESR* erythrocyte sedimentation rate, *hsCRP* high sensitivity C-reactive protein, *BASDAI* Bath Ankylosing Disease Activity Index, *ASDAS* Ankylosing Spondylitis Disease Activity Score, *BASFI* Bath Ankylosing Spondylitis Functional Index, *BASMI* Bath Ankylosing Spondylitis Metrology Index, *BASDAI question (QN)1* overall level of fatigue/tiredness, *BASDAI QN4* overall level of discomfort from areas tender to touch, *mSASSS* Modified Stoke Ankylosing Spondylitis Spinal Score, *mSASSS cervical* Cervical portion of mSASSS, *csDMARD* conventional synthetic disease modifying anti-rheumatic drug, *b* biologic *NSAID* non-steroidal anti-inflammatory drug

^a^*n* = 350, ^b^*n* = 352, ^c^*n* = 351 ^d^*n* = 218, ^e^*n* = 132, ^f^*n* = 217, ^g^*n* = 219

### Factors that influence physical function measured by BASFI

As seen in model 1, female sex had a negative impact on BASFI when introduced as a covariate beside other identified predictors of the outcome (Table 2). Furthermore, in model 2 (All patients), BASDAI QN1 (fatigue) and BASDAI QN4 (Tenderness) were added as independent variables, and now, female sex was no longer a significant independent predictor of BASFI. When comparing models including BASMI to those including mSASSS, BASMI produced consistently higher *R*^2^ values compared to mSASSS. In the best-performing model, including all patients (model 3, *R*^2^ = 0.61), five covariates were independently associated with the BASFI score. Increasing age, higher ASDAS-CRP, BASMI, BASDAI QN1, and BASDAI QN4 all corresponded to a higher BASFI. Furthermore, in the models separated by sex, mSASSS turned out a significant predictor of BASFI in males but not in females. BASDAI QN4 was not associated with BASFI among males after adjusting for BASMI, while in females, this variable remained significant in all models.

**Table 2:** Multivariable linear regression models investigating influencing factors on BASFI in patients with r-axSpA

	Model 1 All patients		Model 2 All patients (mSASSS)		Model 3 All patients (BASMI)		Model 2, Males (mSASSS)		Model 3, Males (BASMI)		Model 2, Females (mSASSS)		Model 3, Females (BASMI)	
*R*^2^	0.53		0.58		0.61		0.62		0.63		0.54		0.60	
	***B*** (95% CI)	***p*-value**	***B*** (95% CI)	***p*-value**	***B*** (95% CI)	***p*-value**	***B*** (95% CI)	***p*-value**	***B*** (95% CI)	***p*-value**	***B*** (95% CI)	***p*-value**	***B*** (95% CI)	***p*-value**
Constant	− 4.02 (− 5.56 to − 2.48)	< 0.001	− 3.89 (− 5.40 to − 2.39)	< 0.001	− 3.56 (− 5.00 to − 2.11)	< 0.001	− 2.85 (− 4.46 to − 1.23)	< 0.001	− 2.90 (− 4.62 to − 1.34)	< 0.001	− 4.62 (− 7.18 to − 2.06)	< 0.001	− 3.77 (− 6.15 to − 1.38)	**0.002**
Age, years	0.03 (0.01 to 0.05)	**0.003**	0.06 (0.04 to 0.08)	** < 0.001**	0.03 (0.01 to 0.05)	** < 0.001**	0.05 (0.03 to 0.07)	** < 0.001**	0.03 (0.01 to 0.06)	**0.008**	0.08 (0.05 to 0.11)	** < 0.001**	0.03 (− 0.01 to 0.07)	0.09
BMI, kg/m^2^	0.05 (0.01 to 0.10)	**0.022**	0.05 (0.01 to 0.09)	**0.024**	0.04 (− 0.00 to 0.08)	0.065	0.04 (− 0.01 to 0.09)	0.093	0.04 (− 0.01 to 0.08)	0.16	0.05 (− 0.03 to 0.13)	0.21	0.04 (− 0.04 to 0.11)	0.35
ASDAS CRP, score	1.73 (1.48 to 1.98)	** < 0.001**	1.04 (0.74 to 1.34)	** < 0.001**	1.03 (0.75 to 1.32)	** < 0.001**	0.84 (0.48 to 1.20)	** < 0.001**	0.84 (0.49 to 1.19)	** < 0.001**	1.39 (0.86 to 1.93)	** < 0.001**	1.36 (0.86 to 1.86)	** < 0.001**
BASMI, score	0.58 (0.41 to 0.74)	** < 0.001**	N/A		0.57 (0.42 to 0.72)	** < 0.001**	NA		0.52 (0.35 to 0.69)	** < 0.001**	NA		0.75 (0.43 to 1.06)	** < 0.001**
mSASSS, score	NA		0.03 (0.02 to 0.04)	** < 0.001**	NA		0.03 (0.02 to 0.05)	** < 0.001**	N/A		0.02 (− 0.01 to 0.06)	0.12	NA	
Fatigue, score	NA		0.34 (0.24 to 0.43)	** < 0.001**	0.32 (0.23 to 0.42)	** < 0.001**	0.41 (0.29 to 0.53)	** < 0.001**	0.41 (0.29 to 0.52)	** < 0.001**	0.27 (0.11 to 0.42)	**0.001**	0.22 (0.08 to 0.37)	**0.003**
Tenderness, score	NA		0.17 (0.07 to 0.26)	**0.001**	0.13 (0.04 to 0.22)	**0.006**	0.14 (0.02 to 0.27)	**0.027**	0.10 (− 0.02 to 0.22)	0.10	0.18 (0.02 to 0.34)	**0.027**	0.15 (0.01 to 0.29)	**0.04**
Female sex	0.62 (0.17 to 1.06)	**0.006**	0.28 (− 0.17 to 0.74)	0.22	0.202 (− 0.22 to 0.62)	0.34	NA		NA		NA		NA	

The outcome of the models is 10 percentiles of Bath Ankylosing Spondylitis Functional Index. Models 1–3 include all patients; models 2–3 are consecutively performed in the cohort separated by sex. Highlighted in bold are *p*-values ≤ 0.05

*BASFI* Bath Ankylosing Spondylitis Functional Index, *r-axSpA* radiographic axial spondyloarthritis, *R*^*2*^ coefficient of determination, *B* unstandardized regression coefficient, *CI* confidence interval, *BMI* body mass index, *ASDAS* Ankylosing Spondylitis Disease Activity Score, *CRP* C-reactive protein, *BASMI* Bath Ankylosing Spondylitis Metrology Index, *mSASSS* Modified Stoke Ankylosing Spondylitis Spinal Score, *Fatigue* Bath Ankylosing Spondylitis Disease Activity Index (BASDAI) question (QN)1, *Tenderness* BASDAI QN4

### Individual BASFI questions and relation to sex

**Fig. 2:**
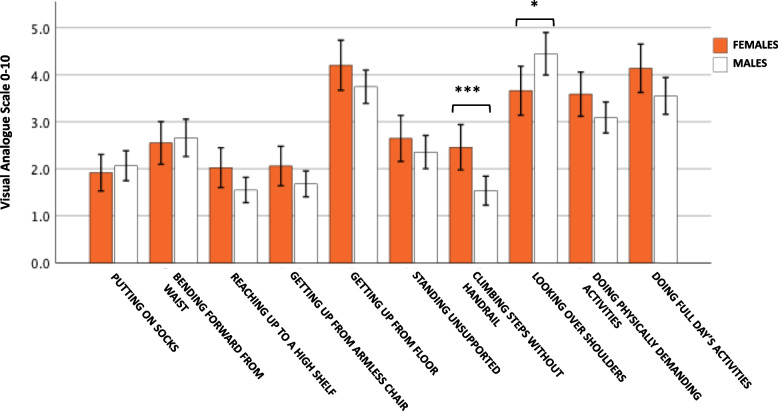
Comparison between the different BASFI questions in males and females with r-axSpA. Values are mean. Independent samples *t*-test was used. Error bars are 95% confidence interval. *p = 0.05, ***p < 0.001. *BASFI* Bath Ankylosing Spondylitis Functional Index; *r-axSpA* radiographic axial spondyloarthritis

Male and female r-axSpA patients were compared regarding individual BASFI questions, presented in Fig. 2. The numerically highest/worst scored question among males was “look over your shoulder without turning the body” (BASFI QN8). The hardest task for females was “getting up from lying on the floor on the back” (BASFI QN5). Significant sex differences were detected in two of the questions; BASFI QN8 was scored worse in males vs females (mean 4.43 ± 3.37 vs 3.74 ± 3.06, *p* = 0.05), whereas “climbing 12–15 steps without using a handrail or walking aid” (BASFI QN7) was scored worse in females vs males (mean 2.49 ± 2.77 vs 1.54 ± 2.32, *p* < 0.001).

### Factors that influence sex differences in individual BASFI questions

Using the ability to look over the shoulder (BASFI QN8) as the outcome in a multivariable linear regression model, age, mSASSS, and ASDAS-CRP were significant independent predictors. Male sex, however, was no longer independently associated with BASFI QN8 after adjusting for mSASSS. In sex-separated models, results were similar; age, mSASSS, and ASDAS-CRP were all significantly associated with the outcome (Table 3). A switch of mSASSS to BASMI improved the female model (*R*^2^ = 0.29 vs. 0.41) but not the male counterpart (*R*^2^ = 0.47 vs. 0.45) (Supplementary Table 2, Additional file 2).

**Table 3:** Multivariable linear regression analyses exploring factors associated with BASFI QN8 and BASFI QN7

	**All**		**Males**		**Females**	
	***B*** (95% CI)	***p*-value**	***B*** (95% CI)	***p*-value**	***B*** (95% CI)	***p*-value**
BASFI QN8; the ability to look over shoulder						
*R*^2^	0.41		0.47		0.29	
Constant	− 0.70 (− 1.97 to 057)	0.31	− 0.31 (− 1.66 to 1.05)	0.70	− 1.08 (− 3.22 to 1.06)	0.32
Age, years	0.06 (0.04 to 0.08)	** < 0.001**	0.05 (0.03 to 0.08)	** < 0.001**	0.07 (0.03 to 0.10)	** < 0.001**
ASDAS CRP, score	0.98 (0.70 to 1.25)	** < 0.001**	0.94 (0.61 to 1.27)	** < 0.001**	1.07 (0.57 to 1.56)	** < 0.001**
mSASSS, score	0.06 (0.05 to 0.08)	** < 0.001**	0.06 (0.05 to 0.08)	** < 0.001**	0.06 (0.03 to 0.09)	** < 0.001**
Female sex	0.10 (− 0.42 to 0.62)	0.70	NA		NA	
BASFI QN7; the ability to climb stairs without a handrail						
*R*^2^	0.37		0.36		0.35	
Constant	− 1.53 (− 2.45 to − 0.60)	0.001	− 1.10 (− 2.09 to − 1.00)	0.032	− 0.76 (− 2.30 to 0.78)	0.33
Age, years	0.03 (0.01 to 0.04)	** < 0.001**	0.03 (0.01 to 0.04)	** < 0.001**	0.02 (− 0.00 to 0.04)	0.07
ASDAS CRP, score	0.36 (0.17 to 0.54)	** < 0.001**	0.37 (0.15 to 0.59)	**0.001**	0.35 (0.03 to 0.67)	**0.034**
BMI, kg/m^2^	0.03 (0.01 to 0.06)	**0.022**	0.03 (0.00 to 0.06)	**0.040**	0.02 (− 0.03 to 0.07)	0.33
Fatigue, score	0.10 (0.04 to 0.16)	**0.001**	0.11 (0.03 to 0.18)	**0.006**	0.09 (− 0.01 to 0.18)	0.08
Tenderness, score	0.08 (0.02 to 0.14)	**0.007**	0.06 (− 0.02 to 0.13)	0.17	0.11 (0.01 to 0.20)	**0.026**
BASMI, score	0.09 (− 0.00 to 0.19)	0.059	0.05 (− 0.06 to 0.16)	0.36	0.22 (0.02 to 0.42)	**0.033**
Female sex	0.40 (0.13 to 0.67)	**0.003**	NA		NA	

The outcomes of the models are 10 percentiles of Bath Ankylosing Spondylitis Functional Index (BASFI) question (QN) 8 and 20 percentiles of BASFI QN7. All patients are included in the first model and consecutively separated by sex. Highlighted in bold are *p*-values ≤ 0.05. BMI, BASDAI QN1, or BASDAI QN4 had no significant independent influence on BASFI QN8 and were excluded from the models. Likewise, mSASSS had no independent influence on BASFI QN7 in any model and was therefore excluded. *R*^*2*^, coefficient of determination; *B*, unstandardized regression coefficient; *CI*, confidence interval; *ASDAS*, Ankylosing Spondylitis Disease Activity Score; *CRP*, C-reactive protein; *mSASSS*, Modified Stoke Ankylosing Spondylitis Spinal Score; *BMI*, body mass index; *Fatigue*, Bath Ankylosing Disease Activity Index (BASDAI) question (QN)1; *Tenderness*, BASDAI QN4; *BASMI*, Bath Ankylosing Spondylitis Metrology Index

Influencing factors of the ability to climb stairs without a handrail (BASFI QN7) were also explored using multivariable linear regression analyses (Table 3). Since mSASSS did not independently predict BASFI QN7 in any of the models, BASMI was used instead (Supplementary Table 2, Additional file 2). In the model including all patients, female sex remained a significant independent predictor of the outcome even after adjusting for age, BMI, ASDAS CRP, BASDAI QN1, BASDAI QN4, and BASMI. In the models separated by sex, BASFI QN7 in males was associated with age, BMI, ASDAS CRP, and BASDAI QN1, while the outcome in females was related to ASDAS CRP, BASDAI QN4, and BASMI. Neither patient- nor physician-reported history of hip arthritis was independently associated to BASFI QN7, in any model.

**Fig. 3:**
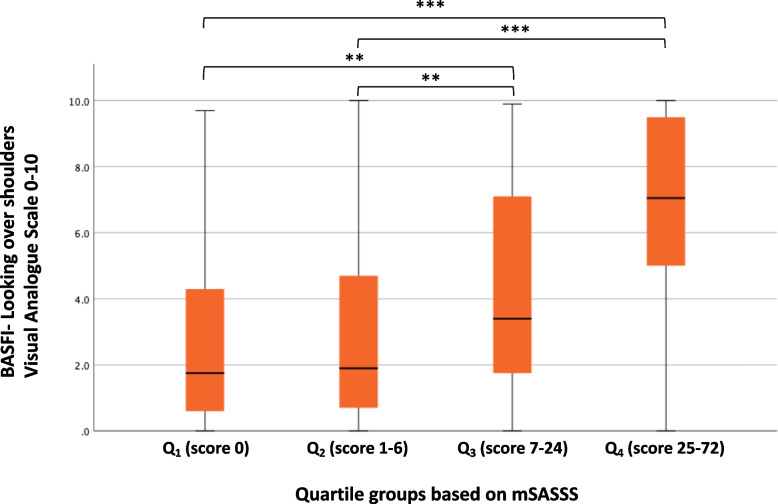
The reported ability to look over the shoulder in r-axSpA patients in relation to mSASSS. BASFI question 8, the ability to look over the shoulder, was examined in the cohort divided into quartiles based on mSASSS. ANOVA and the Tukey HSD/LSD test were used. The number of patients in this analysis was 351. ***p < 0.001, **p < 0.01. *BASFI* Bath Ankylosing Spondylitis Functional Index, *r-axSpA* radiographic axial spondyloarthritis; *mSASSS* Modified Stoke Ankylosing Spondylitis Spinal Score; *Q* Quartile

In consecutive bivariate correlation analyses, BASFI QN8 showed a moderate correlation to mSASSS (*r* = 0.53, *p* = 0.001) and also to the cervical portion of mSASSS (*r* = 0.53, *p* < 0.001) whereas a strong correlation was seen between BASFI QN8 and BASMI (*r* = 0.62, *p* < 0.001) (Supplementary Table 3, Additional file 3). All other BASFI questions correlated weakly to the radiographical indexes, whereas weak to moderate correlation was seen in regard to BASMI. Further analysis on BASFI QN8 was performed by dividing the full cohort in mSASSS quartiles (Fig. 3). Here, significant differences were seen between the quartile-groups in regard to BASFI QN8.

### Factors that influence physical function measured by BASFI in two geographically different r-axSpA cohorts

As a sensitivity analysis, and to investigate potential differences between two geographically distinct r-axSpA cohorts in Sweden, influencing factors of BASFI were also explored in the separated cohorts. Hence, multivariable linear regression analyses were performed using BASFI as the outcome (Supplementary Table 4, Additional file 4). BASDAI QN4 proved a significant predictor of BASFI in r-axSpA patients from western Sweden, but not in participants from northern Sweden. Otherwise, no significant difference between the two cohorts was seen in the models.

## Discussion

In this cross-sectional cohort study, the demographic and disease-related factors that influenced the impairment of physical function assessed by BASFI in r-axSpA patients overall and stratified by sex were investigated. Identified factors contributing to higher BASFI in age, BMI, and sex-adjusted models were ASDAS-CRP, BASMI, mSASSS, fatigue, and discomfort from tender areas. While there was no sex difference in total BASFI scores, females compared to males reported worse function in BASFI QN7, the ability to climb stairs, whereas males compared to females reported worse BASFI QN8, the ability to look over the shoulder without turning the body. When comparing two geographically distinct cohorts, comparable findings were made regarding factors associated with BASFI.

A consistent difference in BASFI scores between male and female r-axSpA patients is not seen in the literature. Some researchers found worse outcomes among females while others reported no difference [12, 25–28]. The sex comparison regarding the total BASFI score in the present study is in line with the latter. We interpret this discrepancy in the literature to be explained by the varying degree of influencing disease-related factors seen in different study cohorts. Indeed, in a study of early axSpA, sex difference regarding BASFI was seen in the non-r-axSpA fraction but not in r-axSpA patients [15]. Among the known determinants of BASFI are disease-related structural alterations of the spine which have been repeatedly shown to associate with BASFI [3, 28–30].

In the present study, surprisingly, no independent effect of mSASSS was seen on BASFI in female patients. Whereas studies specifically investigating the effects of mSASSS on BASFI in females using multivariable models is overall lacking, Lee et al. found no difference between the sexes when comparing bivariate correlations between structural changes of the spine and BASFI [31]. We interpret the present finding as a result of a combination of low levels of spinal radiographic changes in the female r-axSpA patients in our study and strong influence on BASFI by other disease-related factors on the r-axSpA females included in our models.

Furthermore, the independent influence of BASMI and ASDAS-CRP on BASFI was seen regardless of sex, which is consistent with previous studies [30, 32]. In our first model including BASMI, ASDAS-CRP, age, and BMI, female sex independently predicted worse BASFI. After the addition of the two BASDAI questions, fatigue (BASDAI QN1) and discomfort from tender areas (BASDAI QN4), the negative influence of female sex was diminished. This result is in part coherent with previous studies showing that sex does not affect BASFI after adjusting for the total BASDAI score [28, 30]. Furthermore, the findings that BASDAI QN1 and QN4 influence BASFI, independently of disease activity measured by ASDAS-CRP, are in line with what is known about the impact of comorbid fibromyalgia on BASFI [33, 34]. BASDAI QN4 was not scored as high among the males compared to females in our study, which most likely explains the absence of BASDAI QN4 influence on BASFI in the male patients.

To the best of our knowledge, this study is the first to investigate the relationship between sex and separate BASFI questions in a western European r-axSpA cohort. The ability to look over shoulders without turning the body (BASFI QN8) was scored significantly worse among males compared to females. This novel finding was related to the higher level of structural alterations in the spine in males compared to females. Indeed, our results altogether indicate a strong influence of mSASSS on this specific PROM and this impact was seen in both sexes. A previous study of an Iranian r-axSpA cohort did not detect a sex difference regarding BASFI QN8. This discrepancy likely reflects a shorter disease duration of patients and a lower percentage of women seen in the Iranian study compared to the present Swedish cohort [35]. We found that the ability to climb stairs without a handrail (BASFI QN7) was worse in females compared to males, which is, however, in line with the findings of Shalaee et al. [35]. In our study, the influencing factors of BASFI QN7 were indeed different in males vs females. However, because the independent association of female sex and BASFI QN7 persisted despite adjustment for other demographic and disease-related factors in the model, we interpret these findings do not fully explain why BASFI QN7 is worse in females. Plausible contributing factors might be more frequent enthesitis at the greater trochanter in females and lesser muscle mass in the lower extremities [36, 37].

As our secondary aim, the multivariable regression model was repeated, using the BASFI score as the outcome in patients separated by geographical region. The results were essentially consistent in the two separated cohorts from northern Sweden and western Sweden. BASDAI QN4 did not independently influence patients from northern Sweden, which may be an effect of fewer patients in this cohort. Except for BMI which independently associated with BASFI in the combined cohort only, results were similar to the main findings which highlights the robustness of the results.

We interpret the similar BASFI scores in male and female r-axSpA patients found in our study to be explained by equalization of the greater level of structural changes of the spine in males, by the higher level of experienced fatigue and pain in females. Our results emphasize the impact of fatigue on physical function in both men and women, which needs to be acknowledged when assessing r-axSpA patients. Furthermore, since ASDAS-CRP does not measure fatigue, the functional capacity of patients may be overestimated by clinicians focusing, in particular, on disease activity. This stresses the importance of using a dedicated PROM for physical function. Moreover, a low grade of disease-related alterations in the spine does not necessarily correspond to low BASFI, especially in females. Furthermore, our results indicate that self-reported stiffness in the cervical spine is specifically influenced by disease-related structural changes; hence, special attention is warranted in both male and female r-axSpA patients who present in the clinic with such complaints.

Limitations of the study include the cross-sectional nature of data, which introduces some level of uncertainty. Furthermore, in the current analysis, we lack data on radiographically graded sacroiliitis, which has been found to affect BASFI to some degree in a previous study [38]. Fibromyalgia was not a registered co-morbidity in the study. Hence, we could not adjust for this disorder, and because of its known influence on BASFI scores, we consider this a limitation of the study [33]. Finally, the lack of x-rays of the hips is a limitation of the investigation of the ability to climb stairs. The strength of the study is the access to highly detailed data achieved from a well-defined r-axSpA cohort. Moreover, this cohort possesses a relatively high proportion of females, which enables studies on the relationship to sex. Furthermore, patients were included in two geographically distinct regions in Sweden, which promotes generalizability.

In the present study, BASFI QN8 excelled as a strong patient-reported indicator of accrued damage in the spine. Future studies may elucidate whether this PROM also associate with lesions on MRI indicative of active disease or predict structural radiographic progression of the spine. An easily available patient-reported indicator of disease would be useful above all in the early phases of disease and potentially in non-r-axSpA. Moreover, the influencing factors of the reported ability to climb stairs in female r-axSpA patients need to be further clarified. Studies specifically focusing on patterns of physical activity, biomechanics, and factors affecting the lower limbs may disentangle this ambiguity.

## Conclusions

No difference between male and female r-axSpA patients was seen in BASFI scores despite significant sex differences in BASMI, mSASSS, and CRP levels. Our results underline the impact of fatigue and tenderness on BASFI which needs to be acknowledged when assessing physical function in r-axSpA patients. The ability to look over the shoulder was worse in males than females and closely related to spinal mobility and structural spinal changes. Thus, special attention is warranted to r-axSpA patients who bring forward complaints indicative of impaired ability to look over the shoulder.

### Supplementary Information

**Additional file 1:** **Supplementary Table 1.** Correlation matrix between demographic and disease related outcomes in radiographic axial spondyloarthritis.

**Additional file 2:** **Supplementary Table 2.** Multivariable linear regression analyses exploring factors associated with BASFI QN8 and BASFI QN7. BASMI was used instead of mSASSS in the BASFI QN8 model, mSASSS was used instead of BASMI in the BASFI QN7 model.

**Additional file 3:** **Supplementary Table 3.** Correlations between individual BASFI questions and mSASSS, the cervical portion of mSASSS, and BASMI in r-axSpA patients.

**Additional file 4:** **Supplementary Table 4.** Multivariable linear regression models investigating influencing factors of BASFI in two geographically separated cohorts of r-axSpA patients.

## Data Availability

The datasets analyzed during the current study are not publicly available due to the Swedish legislation (the Personal Data Act), but a limited and fully anonymized data set that supports the main analyses is available from the corresponding author on request.

## Abbreviations

AS: Ankylosing spondylitis
ASAS-OMERACT: Assessment of SpondyloArthritis international Society-Outcomes Measures in Rheumatology
ASDAS: Ankylosing Spondylitis Disease Activity Score
B: Unstandardized regression coefficient
b: Biologic
bDMARDs: Biological disease- modifying anti-rheumatic drugs
BASDAI: Bath Ankylosing Disease Activity Index
BASDAI: Question 1 Overall level of fatigue/tiredness
BASDAI: Question 4 Discomfort from areas tender to touch
BASFI: Bath Ankylosing Spondylitis Functional Index
BASFI: Question 5 Getting up from lying on the floor on the back
BASFI: Question 7 Climbing 12–15 steps without using a handrail or walking aid
BASFI: Question 8 Look over your shoulder without turning the body
BASMI: Bath Ankylosing Spondylitis Metrology Index
BMI: Body mass index
CI: Confidence interval
CRP: C-reactive protein
csDMARD: Conventional synthetic disease modifying anti-rheumatic drug
ESR: Erythrocyte sedimentation rate
HLA-B27: Human leukocyte antigen B27
HRQoL: Health-related quality of life
hsCRP: High sensitivity C-reactive protein
mSASSS: Modified Stoke Ankylosing Spondylitis Spinal Score
mSASSS: *Cervical* Cervical portion of the Modified Stoke Ankylosing Spondylitis Spinal Score
NSAID: Non-steroidal anti-inflammatory drug
NUS: University Hospital in Umeå
PROMS: Patient-reported outcome measures
*R*^2^: Coefficient of determination
r-axSpA: Radiographic axial spondyloarthritis
SD: Standard deviation
Q: Quartile
QN: Question

## References

[1] Exarchou S, Lindstrom U, Askling J, Eriksson JK, Forsblad-d'Elia H, Neovius M, Turesson C, Kristensen LE, Jacobsson LT (2015). The prevalence of clinically diagnosed ankylosing spondylitis and its clinical manifestations: a nationwide register study. Arthritis Res Ther.

[2] Webers C, Ramiro S, Landewé R, van der Heijde D, van den Bosch F, Dougados M, van Tubergen A, Boonen A (2018). Sick leave and its predictors in ankylosing spondylitis: long-term results from the Outcome in Ankylosing Spondylitis International Study. RMD Open.

[3] Landewe R, Dougados M, Mielants H, van der Tempel H, van der Heijde D (2009). Physical function in ankylosing spondylitis is independently determined by both disease activity and radiographic damage of the spine. Ann Rheum Dis.

[4] Law L, Beckman Rehnman J, Deminger A, Klingberg E, Jacobsson LTH, Forsblad-d'Elia H (2018). Factors related to health-related quality of life in ankylosing spondylitis, overall and stratified by sex. Arthritis Res Ther.

[5] Calin A, Garrett S, Whitelock H, Kennedy LG, O'Hea J, Mallorie P, Jenkinson T (1994). A new approach to defining functional ability in ankylosing spondylitis: the development of the Bath Ankylosing Spondylitis Functional Index. J Rheumatol.

[6] Ruof J, Sangha O, Stucki G (1999). Comparative responsiveness of 3 functional indices in ankylosing spondylitis. J Rheumatol.

[7] Cronstedt H, Waldner A, Stenstrom CH (1999). The Swedish version of the Bath ankylosing spondylitis functional index. Reliability and validity. Scand J Rheumatol Suppl..

[8] Navarro-Compán V, Boel A, Boonen A, Mease PJ, Dougados M, Kiltz U, et al. Instrument selection for the ASAS core outcome set for axial spondyloarthritis. Ann Rheum Dis. 2023;82(6):763–72. 10.1136/annrheumdis-2022-222747. Epub 2022 Jun 9. 10.1136/annrheumdis-2022-222747 35680390

[9] Wright GC, Kaine J, Deodhar A (2020). Understanding differences between men and women with axial spondyloarthritis. Semin Arthritis Rheum.

[10] Deminger A, Klingberg E, Geijer M, Gothlin J, Hedberg M, Rehnberg E, Carlsten H, Jacobsson LT, Forsblad-d'Elia H (2018). A five-year prospective study of spinal radiographic progression and its predictors in men and women with ankylosing spondylitis. Arthritis Res Ther.

[11] Blasco-Blasco M, Castrejon I, Jovani V, Pascual E, Ruiz-Cantero MT (2021). Reviewing disease activity indices in spondyloarthritis from the sex perspective: a systematic review and metaanalysis. J Rheumatol.

[12] Webers C, Essers I, Ramiro S, Stolwijk C, Landewe R, van der Heijde D, van den Bosch F, Dougados M, van Tubergen A (2016). Gender-attributable differences in outcome of ankylosing spondylitis: long-term results from the Outcome in Ankylosing Spondylitis International Study. Rheumatology (Oxford).

[13] van der Horst-Bruinsma IE, Zack DJ, Szumski A, Koenig AS (2013). Female patients with ankylosing spondylitis: analysis of the impact of gender across treatment studies. Ann Rheum Dis.

[14] Roussou E, Sultana S (2011). Spondyloarthritis in women: differences in disease onset, clinical presentation, and Bath Ankylosing Spondylitis Disease Activity and Functional indices (BASDAI and BASFI) between men and women with spondyloarthritides. Clin Rheumatol.

[15] Tournadre A, Pereira B, Lhoste A, Dubost JJ, Ristori JM, Claudepierre P, Dougados M, Soubrier M (2013). Differences between women and men with recent-onset axial spondyloarthritis: results from a prospective multicenter French cohort. Arthritis Care Res (Hoboken).

[16] van der Linden S, Valkenburg HA, Cats A. Evaluation of diagnostic criteria for ankylosing spondylitis. A proposal for modification of the New York criteria. Arthritis Rheum. 1984;27(4):361–368. 10.1002/art.1780270401 6231933

[17] Boel A, Molto A, van der Heijde D, Ciurea A, Dougados M, Gensler LS, Santos M-J, De Miguel E, Poddubnyy D, Rudwaleit M (2019). Do patients with axial spondyloarthritis with radiographic sacroiliitis fulfil both the modified New York criteria and the ASAS axial spondyloarthritis criteria? Results from eight cohorts. Ann Rheum Dis.

[18] Forsblad-d'Elia H, Wallberg H, Klingberg E, Carlsten H, Bergfeldt L (2013). Cardiac conduction system abnormalities in ankylosing spondylitis: a cross-sectional study. BMC Musculoskelet Disord.

[19] Forsblad-d'Elia H, Law L, Bengtsson K, Smeds J, Ketonen M, Sundstrom B, Ljung L, Geijer M, Soderberg S, Lindqvist P (2021). Biomechanical properties of common carotid arteries assessed by circumferential 2D strain and beta stiffness index in patients with ankylosing spondylitis. J Rheumatol.

[20] Jenkinson TR, Mallorie PA, Whitelock HC, Kennedy LG, Garrett SL, Calin A. Defining spinal mobility in ankylosing spondylitis (AS). The Bath AS Metrology Index. J Rheumatol. 1994; 21(9):1694–1698. 7799351

[21] Sieper J, Rudwaleit M, Baraliakos X, Brandt J, Braun J, Burgos-Vargas R, Dougados M, Hermann KG, Landewe R, Maksymowych W, et al. The Assessment of SpondyloArthritis international Society (ASAS) handbook: a guide to assess spondyloarthritis. Ann Rheum Dis. 2009;68 Suppl 2:ii1–44. 10.1136/ard.2008.104018 19433414

[22] Garrett S, Jenkinson T, Kennedy LG, Whitelock H, Gaisford P, Calin A (1994). A new approach to defining disease status in ankylosing spondylitis: the Bath Ankylosing Spondylitis Disease Activity Index. J Rheumatol.

[23] Lukas C, Landewé R, Sieper J, Dougados M, Davis J, Braun J, Linden Svd, Heijde Dvd, for the Assessment of SpondyloArthritis international S. Development of an ASAS-endorsed disease activity score (ASDAS) in patients with ankylosing spondylitis. Ann Rheum Dis. 2009;68(1):18. 10.1136/ard.2008.094870 18625618

[24] Creemers MC, Franssen MJ, van't Hof MA, Gribnau FW, van de Putte LB, van Riel PL. Assessment of outcome in ankylosing spondylitis: an extended radiographic scoring system. Ann Rheum Dis. 2005;64(1):127-129. 10.1136/ard.2004.020503 PMC1755183 15051621

[25] Taylor AL, Balakrishnan C, Calin A (1998). Reference centile charts for measures of disease activity, functional impairment, and metrology in ankylosing spondylitis. Arthritis Rheum.

[26] Pimentel-Santos FM, Mourão AF, Ribeiro C, Costa J, Santos H, Barcelos A, Pinto P, Godinho F, Cruz M, Vieira-Sousa E, et al. Spectrum of ankylosing spondylitis in Portugal. Development of BASDAI, BASFI, BASMI and mSASSS reference centile charts. Clin Rheumatol. 2012;31(3):447–454. 10.1007/s10067-011-1854-7 22009195

[27] Ward MM, Weisman MH, Davis JC, Reveille JD (2005). Risk factors for functional limitations in patients with long-standing ankylosing spondylitis. Arthritis Rheum.

[28] Boonen A, vander Cruyssen B, de Vlam K, Steinfeld S, Ribbens C, Lenaerts J, Van den Bosch F, Mielants H, Dewulf L, Vastesaeger N. Spinal radiographic changes in ankylosing spondylitis: association with clinical characteristics and functional outcome. J Rheumatol. 2009;36(6):1249-1255. 10.3899/jrheum.080831 19447933

[29] Poddubnyy D, Listing J, Haibel H, Knuppel S, Rudwaleit M, Sieper J (2018). Functional relevance of radiographic spinal progression in axial spondyloarthritis: results from the GErman SPondyloarthritis Inception Cohort. Rheumatology (Oxford).

[30] Machado P, Landewe R, Braun J, Hermann KG, Baraliakos X, Baker D, Hsu B, van der Heijde D (2011). A stratified model for health outcomes in ankylosing spondylitis. Ann Rheum Dis.

[31] Lee W, Reveille JD, Davis JC, Learch TJ, Ward MM, Weisman MH (2007). Are there gender differences in severity of ankylosing spondylitis? Results from the PSOAS cohort. Ann Rheum Dis.

[32] Capelusnik D, Ramiro S, Schneeberger EE, Citera G (2021). Peripheral arthritis and higher disease activity lead to more functional impairment in axial spondyloarthritis: Longitudinal analysis from ESPAXIA. Semin Arthritis Rheum.

[33] Almodovar R, Carmona L, Zarco P, Collantes E, Gonzalez C, Mulero J, Sueiro JL, Gratacos J, Torre-Alonso JC, Juanola X (2010). Fibromyalgia in patients with ankylosing spondylitis: prevalence and utility of the measures of activity, function and radiological damage. Clin Exp Rheumatol.

[34] Azevedo VF, Paiva Edos S, Felippe LR, Moreira RA (2010). Occurrence of fibromyalgia in patients with ankylosing spondylitis. Rev Bras Reumatol.

[35] Shahlaee A, Mahmoudi M, Nicknam MH, Farhadi E, Fallahi S, Jamshidi AR (2015). Gender differences in Iranian patients with ankylosing spondylitis. Clin Rheumatol.

[36] Suetta C, Haddock B, Alcazar J, Noerst T, Hansen OM, Ludvig H, Kamper RS, Schnohr P, Prescott E, Andersen LL (2019). The Copenhagen Sarcopenia Study: lean mass, strength, power, and physical function in a Danish cohort aged 20–93 years. J Cachexia Sarcopenia Muscle.

[37] Segal NA, Felson DT, Torner JC, Zhu Y, Curtis JR, Niu J, Nevitt MC (2007). Greater trochanteric pain syndrome: epidemiology and associated factors. Arch Phys Med Rehabil.

[38] Protopopov M, Sieper J, Haibel H, Listing J, Rudwaleit M, Poddubnyy D (2017). Relevance of structural damage in the sacroiliac joints for the functional status and spinal mobility in patients with axial spondyloarthritis: results from the German Spondyloarthritis Inception Cohort. Arthritis Res Ther.

