# Supplementary material for: Physical function and sex differences in radiographic axial spondyloarthritis: a cross-sectional analysis on Bath Ankylosing Spondylitis Functional Index
Source: Arthritis Res Ther. 2023 Sep 26;25:182. doi: 10.1186/s13075-023-03173-w (PMC10521572; doi:10.1186/s13075-023-03173-w)
Supplement: Supplementary file 1 — Additional file 1: Supplementary Table 1. Correlation matrix between demographic and disease related outcomes in radiographic axial spondyloarthritis. [file 13075_2023_3173_MOESM1_ESM.docx]

**Supplementary Table 1**

| Correlation matrix between demographic and disease related outcomes in radiographic axial spondyloarthritis | | | | | | | | | | | | | | |
| --- | --- | --- | --- | --- | --- | --- | --- | --- | --- | --- | --- | --- | --- | --- |
|  | | | | | | | | | | | | | | |
|  |  | **BASFI** | **Age** | **BMI** | **Symtom**  **duration** | **Delay of**  **diagnosis** | **Smoking**  **packyears** | **BASMI** | **mSASSS** | **ASDAS CRP** | **SJC 66** | **TJC 68** | **BASDAI Q1** | **BASDAI Q4** |
| BASFI, score | r  p Value | 1  NA |  |  |  |  |  |  |  |  |  |  |  |  |
| Age, years | r  p Value | **0.37**  <0.001 | 1  NA |  |  |  |  |  |  |  |  |  |  |  |
| BMI, kg/m^2^ | r  p Value | **0.30**  <0.001 | **0.23**  <0.001 | 1  NA |  |  |  |  |  |  |  |  |  |  |
| Symptom duration, yrs | r  p Value | **0.32**  <0.001 | **0.75**  <0.001 | **0.15**  0.005 | 1  NA |  |  |  |  |  |  |  |  |  |
| Delay of diagnosis, yrs | r  p Value | **0.13**  0.013 | **0.28**  <0.001 | 0.01  0.807 | **0.44**  <0.001 | 1  NA |  |  |  |  |  |  |  |  |
| Smoking packyears | r  p Value | **0.23**  <0.001 | **0.22**  <0.001 | **0.18**  0.001 | **0.12**  0.032 | 0.10  0.066 | 1  NA |  |  |  |  |  |  |  |
| BASMI, score | r  p Value | **0.47**  <0.001 | **0.62**  <0.001 | **0.28**  <0.001 | **0.55**  <0.001 | 0.05  0.377 | **0.26**  <0.001 | 1  NA |  |  |  |  |  |  |
| mSASSS, score | r  p Value | **0.24**  <0.001 | **0.39**  <0.001 | **0.22**  <0.001 | **0.37**  <0.001 | -0.06  0.264 | **0.22**  <0.001 | **0.74**  <0.001 | 1  NA |  |  |  |  |  |
| ASDAS CRP, score | r  p Value | **0.57**  <0.001 | 0.03  0.596 | 0.10  0.055 | 0.06  0.285 | 0.09  0.087 | **0.13**  0.016 | 0.08  0.149 | -0.03  0.638 | 1  NA |  |  |  |  |
| Swollen joint count 66 | r  p Value | 0.06  0.242 | 0.02  0.662 | -0.01  0.904 | 0.08  0.145 | 0.05  0.373 | 0.03  0.575 | 0.02  0.749 | 0.00  0.965 | **0.19**  <0.001 | 1  NA |  |  |  |
| Tender joint count 68 | r  p Value | **0.23**  <0.001 | **0.14**  0.010 | 0.05  0.307 | **0.15**  0.006 | **0.18**  <0.001 | 0.06  0.307 | 0.09  0.097 | **-0.12**  0.030 | 0.07  0.226 | 0.10  0.065 | 1  NA |  |  |
| BASDAI QN1, fatigue | r  p Value | **0.50**  <0.001 | -0.00  0.960 | 0.07  0.186 | 0.08  0.116 | 0.10  0.054 | -0.03  0.582 | 0.02  0.724 | **-0.11**  0.036 | **0.51**  <0.001 | 0.06  0.285 | **0.18**  <0.001 | 1  NA |  |
| BASDAI QN4, tenderness | r  p Value | **0.49**  <0.001 | **0.12**  0.031 | **0.12**  0.021 | **0.14**  0.007 | 0.08  0.131 | -0.01  0.932 | 0.08  0.121 | **-0.15**  0.005 | **0.55**  <0.001 | 0.05  0.387 | **0.25**  <0.001 | **0.48**  <0.001 | 1  NA |
|  |  |  |  |  |  |  |  |  |  |  |  |  |  |  |

Previously known and unknown influencing variables of Bath Ankylosing Functional Index (BASFI). The impact on BASFI of all listed variables was evaluated in subsequent multivariable linear regression models. Except for those included in the correlation matrix, HLA-B27, history of peripheral arthritis, history of hip arthritis, history of anterior uveitis, ever/never smoker were also evaluated in the subsequent models. Highlighted in bold are p-values ≤0.05**.** *r* Pearson correlation coefficient, *BMI* body mass index, *BASMI* Bath Ankylosing Spondylitis Metrology Index, *mSASSS* Modified Stoke Ankylosing Spondylitis Spinal Score, *ASDAS* Ankylosing Spondylitis Disease Activity Score, *CRP* C- reactive protein, *SJC 66* Swollen joint count 66, *TJC 68* Tender joint count 68, *BASDAI question (QN)1* overall level of fatigue/tiredness, *BASDAI QN4* overall level of discomfort from areas tender to touch
