# Supplementary material for: Physical function and sex differences in radiographic axial spondyloarthritis: a cross-sectional analysis on Bath Ankylosing Spondylitis Functional Index
Source: Arthritis Res Ther. 2023 Sep 26;25:182. doi: 10.1186/s13075-023-03173-w (PMC10521572; doi:10.1186/s13075-023-03173-w)
Supplement: Supplementary file 2 — Additional file 2: Supplementary Table 2. Multivariable linear regression analyses exploring factors associated with BASFI QN8 and BASFI QN7. BASMI was used instead of mSASSS in the BASFI QN8 model, mSASSS was used instead of BASMI in the BASFI QN7 model. [file 13075_2023_3173_MOESM2_ESM.docx]

**Supplementary Table 2**

Multivariable linear regression analyses exploring factors associated with BASFI QN8

and BASFI QN7

|  | | | | | | |
| --- | --- | --- | --- | --- | --- | --- |
| BASFI QN8; the ability to look over shoulder (BASMI instead of mSASSS) | | | | | | |
|  | **All** | | **Males** | | **Females** | |
| R^2^ | 0.44 | | 0.45 | | 0.41 | |
|  | **B** (95% CI) | **p-value** | **B** (95% CI) | **p-value** | **B** (95% CI) | **p-value** |
| Constant | -0.35 (-1.60 to 0.89) | 0.58 | -0.80 (-2.16 to 0.57) | 0.25 | -0.58 (-2.53 to 1.38) | 0.56 |
| Age, years | 0.03 (0.00 to 0.05) | **0.024** | 0.04 (0.01 to 0.07) | **0.017** | 0.01 (-0.03 to 0.05) | 0.72 |
| ASDAS CRP, score | 0.83 (0.56 to 1.10) | **<0.001** | 0.82 (0.48 to 1.15) | **<0.001** | 0.83 (0.38 to 1.28) | **<0.001** |
| BASMI, score | 0.89 (0.71 to 1.07) | **<0.001** | 0.81 (0.59 to 1.02) | **<0.001** | 1.10 (0.76 to 1.45) | **<0.001** |
| Female sex | -0.28 (-0.77 to 0.20) | 0.25 | NA |  | NA |  |

|  | | | | | | |
| --- | --- | --- | --- | --- | --- | --- |
| BASFI QN7; the ability to climb stairs without a handrail (mSASSS instead of BASMI) | | | | | |  |
|  | **All** |  | **Males** |  | **Females** |  |
| R^2^ | 0.37 | | 0.36 |  | 0.33 |  |
|  | **B** (95% CI) | **p-value** | **B** (95% CI) | **p-value** | **B** (95% CI) | **p-value** |
| Constant | -1.61 (-2.53 to -0.68) | <0.001 | -1.09 (-2.10 to -0.08) | 0.034 | -1.08 (-2.65 to 0.48) | 0.17 |
| Age, years | 0.03 (0.02 to 0.04) | **<0.001** | 0.03 (0.02 to 0.04) | **<0.001** | 0.04 (0.02 to 0.06) | **<0.001** |
| ASDAS CRP, score | 0.36 (0.18 to 0.54) | **<0.001** | 0.37 (0.15 to 0.59) | **0.001** | 0.36 (0.04 to 0.69) | **0.030** |
| BMI, kg/m^2^ | 0.03 (0.01 to 0.06) | **0.013** | 0.03 (0.00 to 0.07) | **0.035** | 0.03 (-0.02 to 0.08) | 0.23 |
| Fatigue, score | 0.10 (0.04 to 0.16) | **0.001** | 0.11 (0.03 to 0.18) | **0.006** | 0.10 (-0.00 to 0.19) | 0.053 |
| Tenderness, score | 0.08 (0.02 to 0.14) | **0.006** | 0.06 (-0.02 to 0.14) | 0.14 | 0.11 (0.01 to 0.21) | **0.027** |
| mSASSS, score | 0.00 (-0.01 to 0.01) | 0.49 | 0.05 (-0.06 to 0.16) | 0.45 | 0.00 (-0.02 to 0.02) | 0.75 |
| Female sex | 0.39 (0.11 to 0.67) | **0.007** | NA |  | NA |  |
| The outcomes of the models are 10 percentiles of Bath Ankylosing Spondylitis Functional Index (BASFI) question (QN) 8 and 20 percentiles of BASFI QN7. All patients are included in the first model and consecutively separated by sex. Highlighted in bold are p-values ≤0.05. *R^2^* coefficient of determination, *B* unstandardized regression coefficient, *CI* confidence interval, *ASDAS* Ankylosing Spondylitis Disease Activity Score, *CRP* C-reactive protein, *mSASSS* Modified Stoke Ankylosing Spondylitis Spinal Score, *BMI* body mass index, *Fatigue* Bath Ankylosing Disease Activity Index (BASDAI) question (QN)1, *Tenderness* BASDAI QN4 | | | | | | |
