# Supplementary material for: Physical function and sex differences in radiographic axial spondyloarthritis: a cross-sectional analysis on Bath Ankylosing Spondylitis Functional Index
Source: Arthritis Res Ther. 2023 Sep 26;25:182. doi: 10.1186/s13075-023-03173-w (PMC10521572; doi:10.1186/s13075-023-03173-w)
Supplement: Supplementary file 3 — Additional file 3: Supplementary Table 3. Correlations between individual BASFI questions and mSASSS, the cervical portion of mSASSS, and BASMI in r-axSpA patients. [file 13075_2023_3173_MOESM3_ESM.docx]

**Supplementary Table 3**

Correlations between individual BASFI questions and mSASSS, the cervical portion of mSASSS, and BASMI in r-axSpA patients

|  | **mSASSS^a^** | | **mSASSS^a^**  **cervical** |  | **BASMI^b^** |  |
| --- | --- | --- | --- | --- | --- | --- |
|  | **r** | **p- value** | **r** | **p- value** | **r** | **p-value** |
| BASFI QN1 Putting on socks | 0.18 | 0.001 | 0.09 | 0.09 | 0.35 | <0.001 |
| BASFI QN2 Bending forward from waist | 0.19 | <0.001 | 0.12 | 0.026 | 0.36 | <0.001 |
| BASFI QN3 Reaching up to a high shelf | 0.21 | <0.001 | 0.17 | 0.002 | 0.40 | <0.001 |
| BASFI QN4 Getting up from armless chair | 0.05 | 0.40 | 0.01 | 0.806 | 0.25 | <0.001 |
| BASFI QN5 Getting up from floor | 0.21 | 0.001 | 0.14 | 0.01 | 0.44 | <0.001 |
| BASFI QN6 Standing unsupported | 0.07 | 0.17 | 0.05 | 0.347 | 0.25 | <0.001 |
| BASFI QN7 Climbing steps without handrail | 0.06 | 0.25 | 0.04 | 0.440 | 0.28 | <0.001 |
| BASFI QN8 Looking over shoulders | **0.53** | 0.001 | **0.53** | <0.001 | **0.62** | <0.001 |
| BASFI QN9 Doing physically demanding activities | 0.10 | 0.055 | 0.08 | 0.146 | 0.30 | <0.001 |
| BASFI QN10 Doing full day’s activities | 0.07 | 0.17 | 0.04 | 0.461 | 0.28 | <0.001 |
|  |  |  |  |  |  |  |
| ^a^n = 351, ^b^n = 353  Values of BASFI QN8 are marked in bold. *BASMI* Bath Ankylosing Spondylitis Metrology Index, *mSASSS* Modified Stoke Ankylosing Spondylitis Spinal Score**,** *mSASSS cervical* Cervical portion of mSASSS**,** *BASFI* Bath Ankylosing Spondylitis Functional Index, *QN* Question, *r-axSpA* radiographic axial spondyloarthritis, *r* Pearson correlation coefficient | | | | | | |
