# Supplementary material for: Physical function and sex differences in radiographic axial spondyloarthritis: a cross-sectional analysis on Bath Ankylosing Spondylitis Functional Index
Source: Arthritis Res Ther. 2023 Sep 26;25:182. doi: 10.1186/s13075-023-03173-w (PMC10521572; doi:10.1186/s13075-023-03173-w)
Supplement: Supplementary file 4 — Additional file 4: Supplementary Table 4. Multivariable linear regression models investigating influencing factors of BASFI in two geographically separated cohorts of r-axSpA patients. [file 13075_2023_3173_MOESM4_ESM.docx]

**Supplementary Table 4**

Multivariable linear regression models investigating influencing factors of

BASFI in two geographically separated cohorts of r-axSpA patients

|  | | | | |
| --- | --- | --- | --- | --- |
|  | **Western Sweden**, n = 209 | | **Northern Sweden,** n=144 | |
| R^2^ | 0.61 | | 0.57 | |
|  | **B** (95% CI) | **p-value** | **B** (95% CI) | **p-value** |
| Constant | -4.24 (-6.24 to -2.23) | <0.001 | -2.87(-5.23 to -0.51;) | 0.018 |
| Age, years | 0.07 (0.04 to 0.09) | **<0.001** | 0.04 (0.01 to 0.07) | **0.018** |
| BMI, kg/m^2^ | 0.05 (-0.01 to 0.11) | 0.15 | 0.04 (-0.02 to 0.10) | 0.20 |
| ASDAS CRP, score | 1.30 (0.90 to 1.69) | **<0.001** | 0.72 (0.19 to 1.26) | **0.009** |
| mSASSS, score | 0.03 (0.01 to 0.04) | **0.004** | 0.04 (0.02 to 0.05) | **<0.001** |
| Fatigue, score | 0.24 (0.11 to 0.36) | **<0.001** | 0.49 (0.34 to 0.64) | **<0.001** |
| Tenderness, score | 0.16 (0.03 to 0.29) | **0.014** | 0.13 (-0.03 to 0.30) | 0.10 |
| Female sex | 0.19(-0.41 to 0.78) | 0.54 | 0.60 (-0.13 to 1.33) | 0.10 |
|  |  |  |  |  |
| The outcome of the models is 10 percentiles of Bath Ankylosing Spondylitis Functional Index (BASFI). The Cohort is separated into patients from western Sweden and northern Sweden. Highlighted in bold are p-values ≤0.05. *R^2^* coefficient of determination, *B* unstandardized regression coefficient, *CI* confidence interval, *r-axSpA* radiographic axial spondyloarthritis, *BMI* body mass index, *ASDAS* Ankylosing Spondylitis Disease Activity Score, *CRP* C-reactive protein, *BASMI* Bath Ankylosing Spondylitis Metrology Index, *mSASSS* Modified Stoke Ankylosing Spondylitis Spinal Score, *Fatigue* Bath Ankylosing Disease Activity Index (BASDAI) question (QN) 1, Tenderness: BASDAI QN4. | | | | |
